# Comparison of Short-Term DAPT and Long-Term DAPT on the Prognosis of PCI Patients: A Meta-Analysis of Randomized Controlled Trials

**DOI:** 10.31083/j.rcm2310326

**Published:** 2022-09-26

**Authors:** Jiaxin Yang, Yaodong Ding, Rui Wang, Kexin Wang, Xiaoli Liu, Hua Shen, Yan Sun, Hailong Ge, Zhe Fang

**Affiliations:** ^1^Department of Cardiology, Beijing Anzhen Hospital, Capital Medical University, 100029 Beijing, China; ^2^Department of Cardiology, Jiangxi Provincial People's Hospital, The First Affiliated Hospital of Nanchang Medical College, 330006 Nanchang, Jiangxi, China

**Keywords:** dual antiplatelet therapy duration, P2Y12 receptor inhibitor, percutaneous coronary intervention (or PCI), drug-eluting stents (or DES)

## Abstract

**Background:**

Dual antiplatelet therapy (DAPT) is the primary medication for patients after percutaneous coronary intervention (PCI). However, the best DAPT duration is still controversial. This systematic review and meta-analysis aims to assess the safety and effectiveness of short-term (3–6 months) DAPT compared to long-term (12 months) DAPT.

**Methods:**

We searched PubMed, Embase, Cochrane Library, and Web of Science systematically for all the randomized controlled trials (RCTs) which compared the different strategies for DAPT in patients undergoing PCI within ten years prior to January 2021. Major bleeding and any bleeding were identified as the safe endpoints. All causes of death, cardiac death, myocardial infarction, definite/probable stent thrombosis, target vessel revascularization, and stroke were identified as the efficacy endpoints. The hazard ratio (HR) and 95% confidence interval (CI) in each study were abstracted.

**Results:**

Overall, 11 trials and 24,242 patients were included in this meta-analysis with 15-month median follow-up time. Short-term DAPT was related to reduced risks of major bleeding (HR 0.65, 95% CI 0.48–0.89) and any bleeding (HR 0.64, 95% CI 0.53–0.79). No obvious differences in any of the other endpoints were observed. In acute coronary syndrome (ACS) patients with drug-eluting stents (DES), short-term compared with long-term DAPT was related to a decreased risk of major bleeding (HR 0.57, 95% CI 0.37–0.87) without significant increasing in the risks of any bleeding and ischemic endpoints. Furthermore, short-term DAPT followed by P2Y12 receptor inhibitor monotherapy appreciably lowered the risk of major bleeding (HR 0.64, 95% CI 0.42–0.96) and any bleeding (HR 0.58, 95% CI 0.36–0.93). There were no obvious differences concerning death between the different strategies for DAPT.

**Conclusions:**

After PCI with DES, short-term DAPT is safer than long-term DAPT, and is not inferior in effectiveness, even in ACS patients. P2Y12 receptor inhibitor monotherapy following short-term DAPT is also related to a decreased risk of bleeding and may be an alternative anti-platelet strategy.

## 1. Introduction

Dual antiplatelet therapy (DAPT), including aspirin and a P2Y12 receptor inhibitor, is the standard of therapy for patients after percutaneous coronary intervention (PCI) to reduce the risk of stent thrombosis (ST) and prevent coronary atherothrombotic events distal to the stented coronary segment. International guidelines suggest that DAPT should be given for at least 12 months for acute coronary syndromes (ACS) patients with drug-eluting stent (DES); and for patients with stable ischemic heart disease, DAPT should be used for a minimum of 6 months after DES [1, 2]. With the refinements of DES technologies and the emergence of potent P2Y12 receptor inhibitors, the best DAPT duration is still controversial.

The results of several randomized controlled trials (RCTs) had shown that 3–6 months of DAPT after DES had non-inferiority compared with long-term (≥12 months) DAPT [3, 4, 5, 6]. The reason might be that shorter DAPT duration reduced all-cause mortality by reducing bleeding [7], whereas longer DAPT duration was related to a higher risk of any bleeding [8]. However, the risk of myocardial infarction (MI) was raised in 6 months of DAPT, which improved concerns that short-term DAPT might not be safer in ACS patients [9]. A meta-analysis also concluded that 3-month DAPT was related to higher ischemic risk in ACS, although most of the included ACS patients were at comparatively low-risk [10]. However, studies had shown that P2Y12 receptor inhibitor monotherapy after stopping short-term DAPT decreased the bleeding risk without increasing the risk of death, MI, and stroke compared with long-term DAPT [11, 12, 13, 14].

Considering the poor compliance of patients with long-term DAPT and the increasing risk of bleeding, shortening the duration and P2Y12 receptor inhibitor monotherapy may reduce bleeding risks while minimizing atheroembolic events. Therefore, we included the most recent RCTs in our meta-analysis to investigate the differences in the safety and effectiveness between short-term DAPT (3–6 months) and long-term DAPT (12 months) after PCI with DES. Subgroup analyses (ACS and single antiplatelet therapy) were also performed to assess the benefits of P2Y12 receptor inhibitor monotherapy in these patients.

## 2. Materials and Methods

We registered our protocol with PROSPERO (CRD42021260473). This meta-analysis was prepared according to the Preferred Reporting Items for Systematic Reviews and Meta-Analyses (PRISMA) guidelines [15].

### 2.1 Search Strategy

To obtain qualified RCTs, we searched PubMed, Embase, Cochrane Library, and Web of Science for all trials within ten years prior to February 15, 2021, which explored the influence of DAPT duration on the prognosis of PCI patients. The MeSH search terms included the following: Percutaneous Coronary Intervention, Drug-Eluting Stents, Dual Antiplatelet Therapy, Aspirin, Clopidogrel, Prasugrel Hydrochloride, Ticagrelor, and Randomized Controlled Trials. Our search strategies are presented in **Supplementary Detail 1**.

### 2.2 Inclusion/Exclusion Criteria, Outcomes, and Quality Assessment

Trials in line with the following criteria were included: original articles published in English; RCTs comparing different strategies for DAPT in patients undergoing PCI with DES; the duration of short-term DAPT was 3–6 months and the duration of long-term DAPT was 12 months; outcomes included major cardiovascular events and bleeding. We excluded non-RCTs, sub-studies of large studies, and those without the hazard ratio (HR). After removing duplicate articles, the titles and abstracts of the remaining were screened independently by two investigators, and the entire articles were read in detail afterwards to identify trials which met the inclusion criteria. Finally, the data was cross-checked and negotiated to resolve differences.

The prespecified safety endpoints comprised major bleeding and any bleeding. The efficacy endpoints included all causes of death, cardiac death, MI, definite/probable ST, target vessel revascularization (TVR), and stroke. Major bleeding and any bleeding are defined in **Supplementary Tables 1.1** and **1.2**.

Two investigators reviewed the studies, extracted basic information and outcomes independently, and evaluated the included trials for selection bias, performance bias, detection bias, attrition bias, reporting bias, and other sources of biases according to the Cochrane Collaboration Assessment [16] for the risk of bias with Review Manager 5.4.

### 2.3 Statistical Analysis

The data was analyzed by Stata (version 14.2, Stata Corporation, College Station, TX, USA). HR and 95% confidence interval (CI) were abstracted to quantify the effects of different durations. Cochran’s Q and $I^{2}$ was used to assess the heterogeneity. The heterogeneity was regarded as low when the *p* value was >0.10 and the $I^{2}$ < 50%; a fixed-effects model was used when heterogeneity was low. The Egger test and funnel plots were used to complete the bias assessment. Subgroup analyses were also performed in patients with ACS who received short-term DAPT (S-DAPT) and single antiplatelet therapy (SAPT). Sensitivity analyses were also performed.

## 3. Results

### 3.1 Study Characteristics and Bias Assessment

**Fig. 1.**
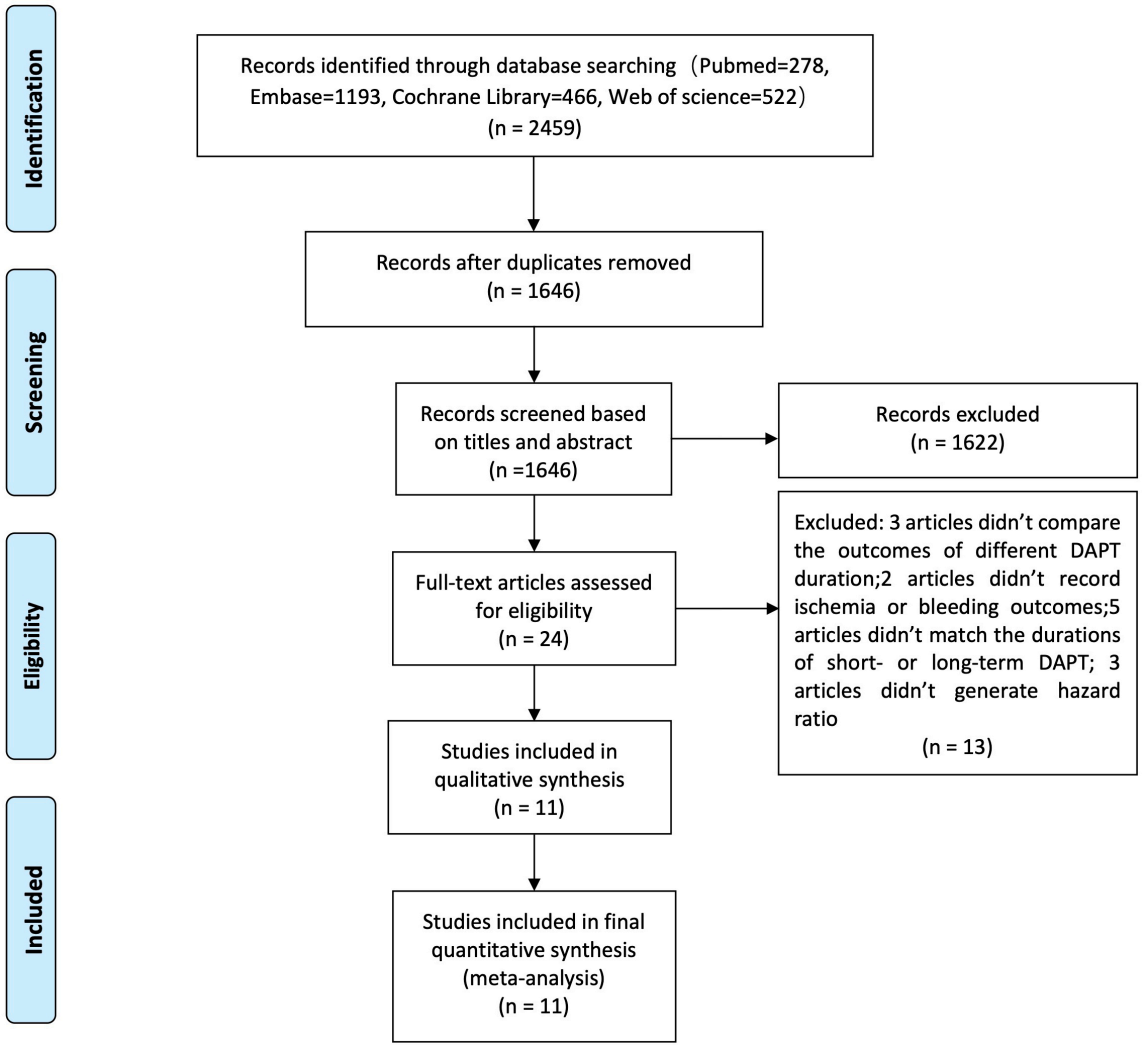
**The selection process included studies**.

Of 2459 articles, 1646 were screened after removing duplications and 1622 articles were ruled out when viewing titles and abstracts. Twenty-four potentially eligible articles were carefully scrutinized for full texts. Finally, a total of 11 trials encompassing 24,242 patients were enrolled in this meta-analysis. Six studies were from Korea, representing approximately 53.5% of the population. Caucasian countries accounted for approximately 46.5% of the patients. The selection process is shown in Fig. 1.

**Fig. 2.**
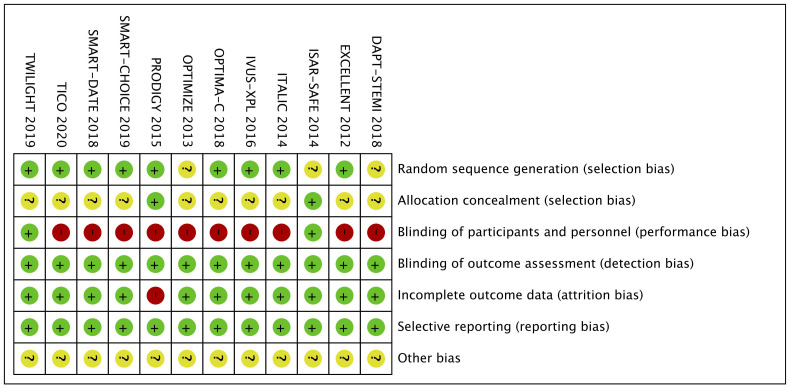
**Quality assessment of included studies**.

For direct comparisons, 7 trials [4, 5, 9, 16, 17, 18, 19] compared 6-month DAPT followed by aspirin monotherapy with 12-month DAPT. Two trials [6, 20] compared 3-month DAPT followed by aspirin monotherapy and 2 trials [14, 21] compared 3 months of DAPT followed by P2Y12 receptor inhibitor monotherapy with 12 months of DAPT. In addition, 5 trials [4, 9, 14, 18, 20] reported outcomes in ACS patients. The median follow-up duration for all trials was 15 (range from 12 to 24) months. Among these 11 trials, 6 trials [5, 6, 16, 17, 18, 19] used aspirin plus clopidogrel as DAPT strategy and continued aspirin monotherapy after stopping short-term DAPT. Four trials [4, 9, 20, 21] used aspirin plus P2Y12 receptor inhibitor (clopidogrel, ticagrelor, or prasugrel) for short and long DAPT, 3 trials [4, 9, 20] of them continued aspirin by stopping P2Y12 receptor inhibitor after short-term DAPT, but 1 trial [21] discontinued aspirin and continued clopidogrel monotherapy. One trial [14] used ticagrelor plus aspirin for DAPT, and ticagrelor monotherapy for SAPT. The baseline characteristics of the included trials and participants are shown in Table 1 (Ref. [4, 5, 6, 9, 14, 16, 17, 18, 19, 20, 21]) and **Supplementary Table 2**. According to the Newcastle-Ottawa Scale, there were 8 trials [5, 9, 14, 16, 18, 19, 20, 21]describing the methods of generating random sequences, such as computer-generated random sequences. Two trials [17, 20] described sequence hid through central allocation. One trial [17] used double-blind methods, and all trials had blinded outcome assessments. There were no incomplete outcome data and selective reporting. Biases from other sources were unknown. The results of the risk bias assessment of each RCT are summarized in Fig. 2.

**Table 1.** **The baseline characteristics of the included trials and participants**.

Trials	Country	Weight	DAPT	Patients	ACS	Clopidogrel	Ticagrelor	Prasugrel	SAPT	Follow-up	Primary endpoint	Secondary endpoints
DAPT-STEMI (2018) [4]	Netherlands, Norway, Poland	870 (3.6%)	6/12-month	433/437	100/100	42.0/42.0	29.0/28.0	29.0/30.0	aspirin	24-month	Composite of all causes of death, MI, any revascularization, stroke, and TIMI Major bleeding	Composite of all causes of death, MI, ST, stroke, and TIMI major bleeding; the individual components of the primary endpoint
EXCELLENT (2012) [16]	Korea	1443 (6.0%)	6/12-month	722/721	51.1/52.0	98.7/99.6	-	-	aspirin	12-month	Composite of cardiac death, MI, or TVR	Cardiac death, MI, TVR, all causes of death, death or MI, ST, TIMI major bleeding, MACCE (composite of death, MI, stroke, or any revascularization), safety endpoint (composite of death, MI, stroke, ST, or TIMI major bleeding)
ISAR-SAFE (2014) [17]	Germany, Belgium, USA	4000 (16.5%)	6/12-month	1997/2003	39.8/40.3	$1.00	-	-	aspirin	15-month	Composite of death, MI, ST, stroke, or TIMI major bleeding	Composite of death, MI, ST, stroke, TIMI major and minor bleeding, BARC bleeding ≥2
ITALIC (2014) [18]	Europe and the Middle East	1822 (7.5%)	6/12-month	912/910	23.1/23.8	98.9/98.4	0.1/-	1.6/1.8	aspirin	12-month	Composite of death, MI, repeat emergency revascularization, stroke, or major bleeding	Composite of death, MI, or repeat emergency revascularization, and stroke requiring readmission
IVUS-XPL (2016) [19]	Korea	1400 (5.8%)	6/12-month	699/701	49.1/48.9	$1.00	-	-	aspirin	12-month	Composite of cardiac death, MI, stroke, or TIMI major bleeding	Individual components of primary outcome
OPTIMA-C (2018) [5]	South Korea	1367 (5.6%)	6/12-month	683/684	50.4/50.9	$1.00	-	-	aspirin	12-month	Composite of cardiac death, MI, or ischemia-driven target lesion revascularization at 12 months	Percentage of uncovered struts at six months
OPTIMIZE (2013) [6]	Brazil	3119 (12.9%)	3/12-month	1563/1556	31.6/32.3	$1.00	-	-	aspirin	12-month	Composite of all cause death, MI, stroke, or major bleeding	ST, target lesion and TVR, MACE (all cause death, MI, emergent CABG surgery, or target lesion revascularization), and any bleeding
REDUCE (2019) [20]	Italy, Netherland, Belgium	1460 (6.0%)	3/12-month	733/727	100/100	41.1/40.5	47.9/41.1	11.1/9.7	aspirin	24-month	Composite of all-cause mortality, MI, definite/probable ST, stroke, TVR, and bleeding (BARC 2–5)	Pre-specified Landmark analysis of primary endpoint from 3 to 12-month, individual components of the primary composite endpoint
SMART-CHOICE (2019) [21]	Korea	2993 (12.3%)	3/12-month	1495/1498	58.2/58.1	76.9/77.6	19.0/17.9	4.1/4.5	P2Y12	12-month	Composite of all-cause death, MI, or stroke	Components of the primary end point and bleeding defined as BARC 2 to 5
SMART-DATE (2018) [9]	Korea	2712 (11.2%)	6/12-month	1357/1355	100/100	79.7/81.8	*	*	aspirin	18-month	Composite of all causes of death, MI, or stroke	Individual components of the primary endpoint, definite/probable ST, BARC type 2–5 bleeding
TICO (2020) [14]	Korea	3056 (12.6%)	3/12-month	1527/1529	100/100	$1.00	-	-	ticagrelor	12-month	Composite of TIMI major bleeding and MACCE (death, MI, ST , stroke, and TVR)	Major bleeding, MACCE, major or minor bleeding, death, MI, ST, stroke, TVR, composite of cardiac death or MI, composite of cardiac death, MI, ST, or TVR

TIMI, Thrombolysis in Myocardial Infarction; BARC, Bleeding Academic Research Consortium; GUSTO, Global Utilization of Streptokinase and TPA for Occluded arteries; MACCE, Major adverse cardiac and cerebrovascular events; MACE, Major adverse cardiovascular events; MI, myocardial infarction; TVR, target vessels revascularization; ST, stent thrombosis; *, It used different P2Y12 receptor inhibitor but didn’t mention the proportion.

### 3.2 Outcomes of Meta-Analysis

Due to the low heterogeneity after testing all endpoints (*p *> 0.10 and $I^{2}$ < 50%), a fixed-effects model was used.

#### 3.2.1 Bleeding Endpoints

**Fig. 3.**
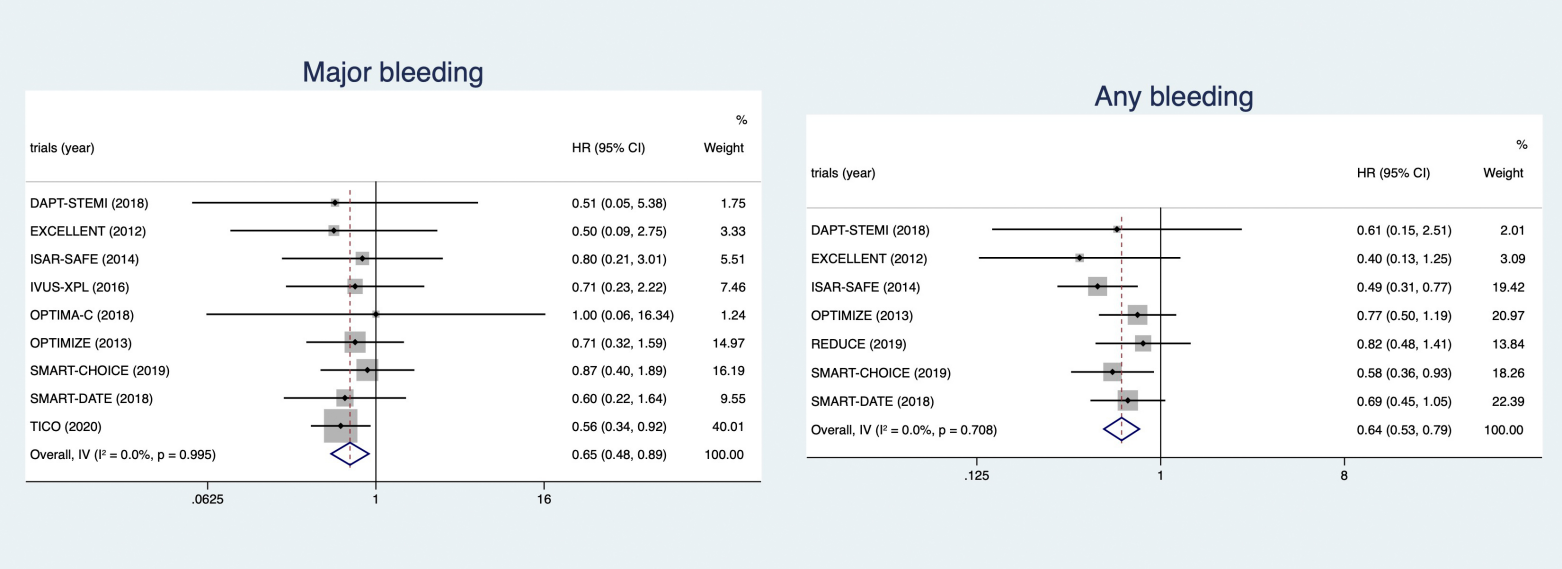
**The forest plots of major bleeding and any bleeding**.

Nine trials recorded major bleeding and 7 trials recorded any bleeding. Short-term DAPT was relevant to reduced risks of major bleeding (HR 0.65, 95% CI 0.48–0.89) and any bleeding (HR 0.64, 95% CI 0.53–0.79) compared with 12-month DAPT. The forest plots of major bleeding and any bleeding are shown in Fig. 3.

#### 3.2.2 Mortality, Ischemia Endpoints, and Stroke 

**Fig. 4.**
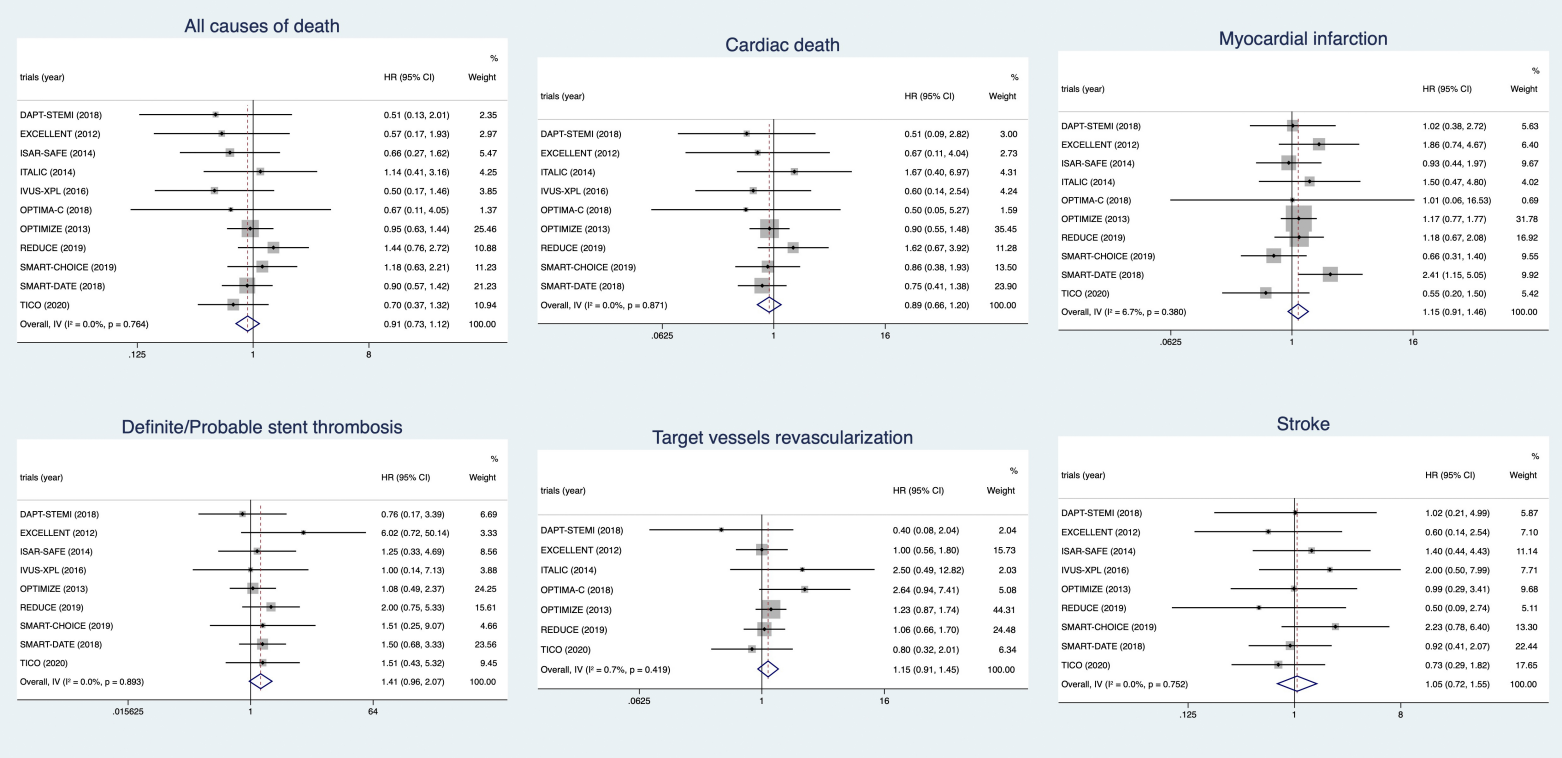
**The forest plots of death, ischemia endpoints, and stroke**.

Eleven trials recorded all causes of death, and 9 trials recorded cardiac death. No differences were observed in the risks of all causes of death (HR 0.91, 95% CI 0.73–1.12) and cardiac death (HR 0.89, 95% CI 0.66–1.20) between different strategies for DAPT. Ten trials recorded MI, 9 trials recorded definite/probable ST, and 7 trials recorded TVR. Compared to 12-month DAPT, short-term DAPT was irrelevant to higher risks of MI (HR 1.15, 95% CI 0.91–1.46), definite/probable ST (HR 1.41, 95% CI 0.96–2.07), and TVR (HR 1.15, 95% CI 0.91–1.45). Nine trials recorded stroke. Compared to 12-month DAPT, short-term DAPT did not increase or decrease the risk of stroke(HR 1.05, 95% CI 0.72–1.55). The forest plots of death, ischemia endpoints, and stroke are shown in Fig. 4.

### 3.3 Subgroup Analysis

Subgroup analyses were performed according to the short-term DAPT duration (S-DAPT), single antiplatelet therapy (SAPT), and ACS (**Supplementary Table 3**). Compared with 12-month DAPT, 3-month DAPT was related to lower risks of major bleeding (HR 0.65, 95% CI 0.45–0.94) and any bleeding (HR 0.71, 95% CI 0.54–0.93), whereas no such benefit in major bleeding was observed with 6-month DAPT. P2Y12 receptor inhibitor monotherapy after short-term DAPT significantly decreased the risks of major bleeding (HR 0.64, 95% CI 0.42–0.96) and any bleeding (HR 0.58, 95% CI 0.36–0.93), but only 1 trial recorded any bleeding. Aspirin after short-term DAPT did not decrease the risk of major bleeding (HR 0.67, 95% CI 0.42–1.08), but was related to a low risk of any bleeding (HR 0.66, 95% CI 0.53–0.82). In patients with ACS, it resulted in a reduced risk of major bleeding (HR 0.57, 95% CI 0.37–0.87) and a non-significant risk of any bleeding (HR 0.73, 95% CI 0.53–1.01) compared with 12-month DAPT. Among these subgroups, different DAPT strategies were not differ significantly with respect to death, and ischemia end and stroke.

### 3.4 Sensitivity Analysis and the Meta-Regression

We evaluated the stability of the outcomes by removing one trial and recombining the remaining trials, then performed a sensitivity analysis for each endpoint. As shown in **Supplementary Table 4**, we obtained similar outcomes, which confirms the stability of our research. No publication bias was found in the funnel plots and Egger tests as shown in **Supplementary Fig. 1** and **Supplementary Table 5**.

## 4. Discussion

In this meta-analysis, we included 11 RCTs and 24,242 patients to assess the safety and effectiveness of short-term and long-term (3–6 months vs 12 months) DAPT among patients who underwent PCI with DES. Compared with 12-month duration of DAPT, short-term DAPT strategies were superior for major bleeding and any bleeding, and non-inferior for all causes of death, cardiac death, MI, definite/probable ST, TVR, and stroke. Even in ACS patients, short-term DAPT continued to be superior in reducing major bleeding. In addition, 3-month DAPT and P2Y12 receptor inhibitor monotherapy after short-term DAPT were associated with lower risks of major bleeding.

Establishing the best strategy of DAPT after DES is crucial to minimize the risk of bleeding and ischemic events. The results of several RCTs demonstrated that short-term (3–6 months) DAPT was non-inferior for the occurrence of death, ischemia, and bleeding among general and ACS patients [4, 6, 16, 20]. A network meta-analysis concluded that 12-month DAPT led to a higher incidence of any bleeding compared to short-term DAPT [8]. Furthermore, subsequent bleeding complications after successful DES implantation were strongly associated with all causes of death, and the magnitude of the effect of bleeding on mortality exceeded that of an MI [22]. Therefore, efforts to reduce the incidence of bleeding after PCI with DES may further improve outcomes in these patients. DES technology is constantly being updated. Compared with bare-metal stents, second-generation DES have been related to a lessening 1-year rate of definite ST [23]; compared with the first-generation DES, it brings out larger stent coverage, less inflammation, fewer fibrin deposits, and less thrombosis [24]. Based on these results, some researchers have questioned whether the DAPT duration should again be shortened.

Our meta-analysis sustained the premise that the DAPT duration may be safely shortened. Short-term DAPT was related to a decreased risk of major bleeding and any bleeding. Moreover, no differences were observed in the incidence of all causes of death, cardiac death, MI, definite/probable ST, TVR, and stroke between different DAPT durations. Therefore, we concluded that short-term DAPT was as effective as 12-month DAPT with a better safety profile. These important findings supported the clinical necessity of defining a new DAPT regimen. Short-term DAPT has a more favorable balance between bleeding and ischemia, regardless of gender [25], age [26], and diabetes [27]. At the same time, clinicians should refer to the recommendations of the European Society of Cardiology guidelines [28] and the 2021 ACC/AHA/SCAI Guideline for Coronary Artery Revascularization [29] to determine individualized risks (low bleeding risk vs high bleeding risk).

For ACS patients, the guideline [29] recommended 12 months of DAPT, which could be extended more than 12 months if they were in low bleeding risk [29], while in patients with higher bleeding risk it should be shorten to 6 months [29]. Scientific societies supported DAPT after ACS based on results from the CURE trial [30]. CURE demonstrated that 3–12 months (mean duration, 9 months) of DAPT reduced the risk of MI and recurrent ischemia and increased the risk of major bleeding in patients with ACS without ST-segment elevation [30]. However, it was conducted 2 decades ago and compared the differences between DAPT and aspirin alone, which supported DAPT per se rather than the duration of 12 months or longer. Newer generation DES technologies have been confirmed to minimize the risks of MI and ST [31, 32]. Moreover, a landmark analysis of this trial demonstrated that DAPT achieved almost all the benefits in the first 3 months after randomization [33]. In recent years, studies on the strategies of DAPT in ACS patients, including RCTs [4, 9, 14, 20] and meta-analyses [10], had shown that short-term DAPT was non-inferior in reducing the occurrence of major bleeding, but no consistent results could be concluded in safety. The main problems were myocardial infarction and stents thrombus [9, 10]. In the multicenter SMART-DATE trial [9], a total of 2712 ACS patients were randomized to 6-month (n = 1357) or 12-month or longer (n = 1355) DAPT. As for major adverse cardiovascular and cerebrovascular events (MACCE), 6-month DAPT was non-inferior to long-term (≥12 months) DAPT, while the incidence of MI was significantly higher [9]. However, there was no obvious difference in ST between the two groups [9]. It was concluded that long-term DAPT might lower the risk of MI by prevention of non-target vessel MI instead of lessening of ST. Similarly, a network meta-analysis [10] found that 3-month instead of 6-month DAPT was related to higher risks of MI and definite/probable ST, compared with 12-month DAPT. However, the number of ACS patients in their study was only 4758, which might have limited the statistical power of the study, and limited the conclusions that could be made. Conversely, no noticeable differences were observed with regard to MI and ST between different durations of DAPT in DAPT-STEMI [4], REDUCE [20] and TICO [14]. Our findings were in line with these studies. In our current meta-analysis, short-term DAPT resulted in an absolute reduction in major bleeding, whereas there were no differences in all causes of death, cardiac death, MI, definite/probable ST, TVR, and stroke among the 8890 ACS patients.

These low event rates might be attributed to the improvements in the design of the second-generation DES, or to the development of atherosclerotic plaques. Compared with stable angina pectoris (SAP), multiple complex coronary plaques are more common and coronary plaques are more unstable in ACS [34]. The unstable plaques are treated during ACS, and the remaining multiple complex lesions are generally treated during subsequent elective PCI. Regarding the unstable plaques, 75% of them seem to stabilize or heal during the 12-month follow-up and 25% remain unchanged [35]. Thus, these plaques are much more likely to maintain clinically silent or present with stable symptoms rather than ACS. DAPT used as secondary prevention may decrease cardiovascular events, but these events are uncommon. The benefits from the reduction of ischemic events by long-term DAPT are not enough to compensate for the increase in bleeding events. In summary, if clinically warranted, short-term DAPT was also feasible and safe even in ACS, especially in those with high bleeding risk.

We conducted subgroup analyses based on the different strategies for DAPT. P2Y12 receptor inhibitor monotherapy after short-term DAPT was related to lower risk of major bleeding compared with 12-month DAPT, with no obvious differences in death, ischemia endpoints, and stroke. However, no such benefit was observed with aspirin monotherapy on major bleeding during the follow-up period. It must be mentioned that in 3 large RCTs [6, 14, 21] which compared 3-month DAPT with 12-month DAPT and recorded major bleeding, 2 [14, 21] of them stopped aspirin after 3-month DAPT and continued P2Y12 receptor inhibitor monotherapy for another 9 months. In the TICO trial of ACS patients, ticagrelor monotherapy brought out a significant 2% absolute reduction in the composite outcome of major bleeding and MACCE, with a significant reduction in major bleeding [14]. In the SMART-CHOICE trial, clopidogrel monotherapy was non-inferior to 12-month DAPT for MACCE and was related to a lower rate of bleeding [21]. The activation of the P2Y12 receptor is the critical part in the production of platelet thromboxane (TX) A2 *in vitro* and *in vivo * [36]. A strong P2Y12 receptor inhibitor alone can block platelet aggregation through the TXA2-dependent pathway, while aspirin has little effect [37]. In the existence of the P2Y12 receptor inhibitor, the additional inhibitory effect of aspirin on platelet aggregation may be minimal. A study has also shown that P2Y12 receptor inhibitor monotherapy and DAPT inhibit the activation of the hemostatic system to the same extent [38]. Therefore, after short-term DAPT, the P2Y12 receptor inhibitor monotherapy may be a suitable antiplatelet strategy to reduce the risk of bleeding in patients with SAP or ACS treated with DES while maintaining anti-ischemic benefits.

A meta-analysis by Li *et al*. [39] reached comparable conclusions to our study; however there were several differences. First, they compared 1–6 months DAPT with ≥12 months DAPT, while we compared 3–6 months DAPT with 12 months DAPT. Second, they extracted risk ratios (RR) and 95% CI. We included the original research results and directly extracted HR and 95% CI. Third, we included the most recent randomized controlled trial TICO [14] and ruled out the studies that accepted other anticoagulant drugs or lacked HR. Finally, we performed a subgroup analysis of ACS patients so that our conclusions could be applied to different populations.

This meta-analysis has several limitations. We included results from first generation DES no longer used in clinical practice. The data to justify shortening the duration of DAPT may be even further strengthened by using only data involving second-generation DES [24]. Finally, all trials included in our meta-analysis are open-label and may lead to bias. In addition, different studies had slightly different definitions of certain clinical endpoints, which may introduce an element of effect modification. The determination of bleeding and bleeding-related deaths is difficult, so these findings should be interpreted with caution. Although the trials we included were multicenter, most of them were from South Korea and Caucasian countries, and there was a lack of relevant data from African countries with predominate black populations. Therefore, more research is needed to confirm the safety and efficacy of different DAPT strategies worldwide.

## 5. Conclusions

Compared with long-term DAPT, short-term DAPT reduced bleeding after PCI with DES and was not inferior in the incidence of ischemic events. Short-term DAPT was also feasible and safely applicable in ACS patients. P2Y12 receptor inhibitor monotherapy after short-term DAPT might be an alternative anti-platelet strategy, and should be further investigated in larger studies.

## Supplementary Material

Supplementary material associated with this article can be found, in the online version, at https://doi.org/10.31083/j.rcm2310326.
