# Supplementary material for: Comparison of Short-Term DAPT and Long-Term DAPT on the Prognosis of PCI Patients: A Meta-Analysis of Randomized Controlled Trials
Source: Rev Cardiovasc Med. 2022 Sep 26;23(10):326. doi: 10.31083/j.rcm2310326 (PMC11267325; doi:10.31083/j.rcm2310326)
Supplement: Supplementary file 1 [file 2153-8174-23-10-326-s1.zip › 2153-8174-23-10-326-s1/Supplementary materials.docx]

# Supplementary detail 1. Search Strategies

**PubMed: (n=278), we used shortcut keys to select RCTs from the recent 10 years**

(((((((((((((("Percutaneous Coronary Intervention"[Mesh]) OR (Coronary Intervention, Percutaneous[Title/Abstract])) OR (Coronary Interventions, Percutaneous[Title/Abstract])) OR (Intervention, Percutaneous Coronary[Title/Abstract])) OR (Interventions, Percutaneous Coronary[Title/Abstract])) OR (Percutaneous Coronary Interventions[Title/Abstract])) OR (Percutaneous Coronary Revascularization[Title/Abstract])) OR (Coronary Revascularization, Percutaneous[Title/Abstract])) OR (Coronary Revascularizations, Percutaneous[Title/Abstract])) OR (Percutaneous Coronary Revascularizations[Title/Abstract])) OR (Revascularization, Percutaneous Coronary[Title/Abstract])) OR (Revascularizations, Percutaneous Coronary[Title/Abstract])) OR (PCI[Title/Abstract])) OR ((((((((((((((("Drug-Eluting Stents"[Mesh]) OR (Drug Eluting Stents[Title/Abstract])) OR (Stents, Drug-Eluting[Title/Abstract])) OR (Stents, Drug Eluting[Title/Abstract])) OR (Drug-Eluting Stent[Title/Abstract])) OR (Drug Eluting Stent[Title/Abstract])) OR (Stent, Drug-Eluting[Title/Abstract])) OR (Drug-Coated Stents[Title/Abstract])) OR (Drug Coated Stents[Title/Abstract])) OR (Stents, Drug-Coated[Title/Abstract])) OR (Stents, Drug Coated[Title/Abstract])) OR (Drug-Coated Stent[Title/Abstract])) OR (Drug Coated Stent[Title/Abstract])) OR (Stent, Drug-Coated[Title/Abstract])) OR (DES[Title/Abstract]))) AND (((((((("Dual Anti-Platelet Therapy"[Mesh]) OR (Anti-Platelet Therapies, Dual[Title/Abstract])) OR (Anti-Platelet Therapy, Dual[Title/Abstract])) OR (Dual Anti Platelet Therapy[Title/Abstract])) OR (Dual Anti-Platelet Therapies[Title/Abstract])) OR (DAPT[Title/Abstract])) OR (DAPT[Title/Abstract])) OR (((((((((((((((((((("Clopidogrel"[Mesh]) OR (SC 25989C[Title/Abstract])) OR (SC 25990C[Title/Abstract])) OR (SR 25989[Title/Abstract])) OR (Clopidogrel-Mepha[Title/Abstract])) OR (Clopidogrel-Mepha[Title/Abstract])) OR (Clopidogrel Sandoz[Title/Abstract])) OR (Iscover[Title/Abstract])) OR (Clopidogrel Napadisilate[Title/Abstract])) OR (Clopidogrel Hydrochloride[Title/Abstract])) OR (PCR 4099[Title/Abstract])) OR (PCR-4099[Title/Abstract])) OR (Clopidogrel Besylate[Title/Abstract])) OR (Clopidogrel Besilate[Title/Abstract])) OR (Clopidogrel, (+)(S)-isomer[Title/Abstract])) OR (Plavix[Title/Abstract])) OR (Clopidogrel Bisulfate[Title/Abstract])) OR ((((((((((((((("Prasugrel Hydrochloride"[Mesh]) OR (Hydrochloride, Prasugrel[Title/Abstract])) OR (Prasugrel HCl[Title/Abstract])) OR (HCl, Prasugrel[Title/Abstract])) OR (CS 747[Title/Abstract])) OR (747, CS[Title/Abstract])) OR (CS-747[Title/Abstract])) OR (CS747[Title/Abstract])) OR (Prasugrel[Title/Abstract])) OR (Efient[Title/Abstract])) OR (Effient[Title/Abstract])) OR (LY 640315[Title/Abstract])) OR (640315, LY[Title/Abstract])) OR (LY640315[Title/Abstract])) OR (LY-640315[Title/Abstract]))) OR ((((((("Ticagrelor"[Mesh]) OR (Brilique[Title/Abstract])) OR (AZD 6140[Title/Abstract])) OR (AZD6140[Title/Abstract])) OR (AZD-6140[Title/Abstract])) OR (Brilinta[Title/Abstract])) OR (3-(7-((2-(3,4-Difluorophenyl)cyclopropyl)amino)-5-(propylthio)-3H-(1-3)-triazolo(4,5-d)pyrimidin-3-yl)-5-(2-hydroxyethoxy)cyclopentane-1,2-diol[Title/Abstract]))) AND ((((((((((((((((((("Aspirin"[Mesh]) OR (Acetylsalicylic Acid[Title/Abstract])) OR (Acid, Acetylsalicylic[Title/Abstract])) OR (2-(Acetyloxy)benzoic Acid[Title/Abstract])) OR (Acylpyrin[Title/Abstract])) OR (Aloxiprimum[Title/Abstract])) OR (Colfarit[Title/Abstract])) OR (Dispril[Title/Abstract])) OR (Easprin[Title/Abstract])) OR (Ecotrin[Title/Abstract])) OR (Endosprin[Title/Abstract])) OR (Magnecyl[Title/Abstract])) OR (Micristin[Title/Abstract])) OR (Polopirin[Title/Abstract])) OR (Polopiryna[Title/Abstract])) OR (Solprin[Title/Abstract])) OR (Solupsan[Title/Abstract])) OR (Zorprin[Title/Abstract])) OR (Acetysal[Title/Abstract]))))

**Web of Science: (n=522)**


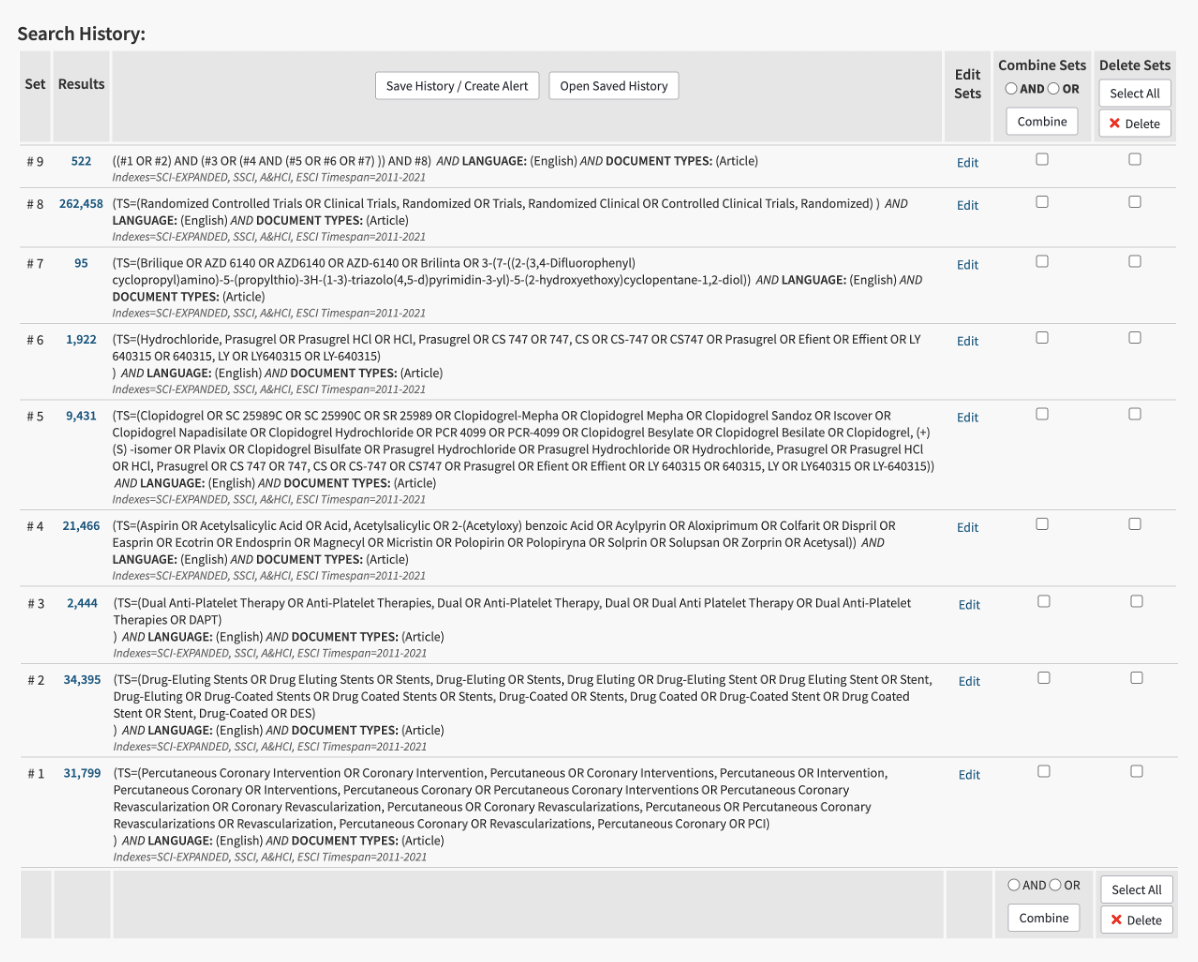


**Cochrane Library: (n=466)**


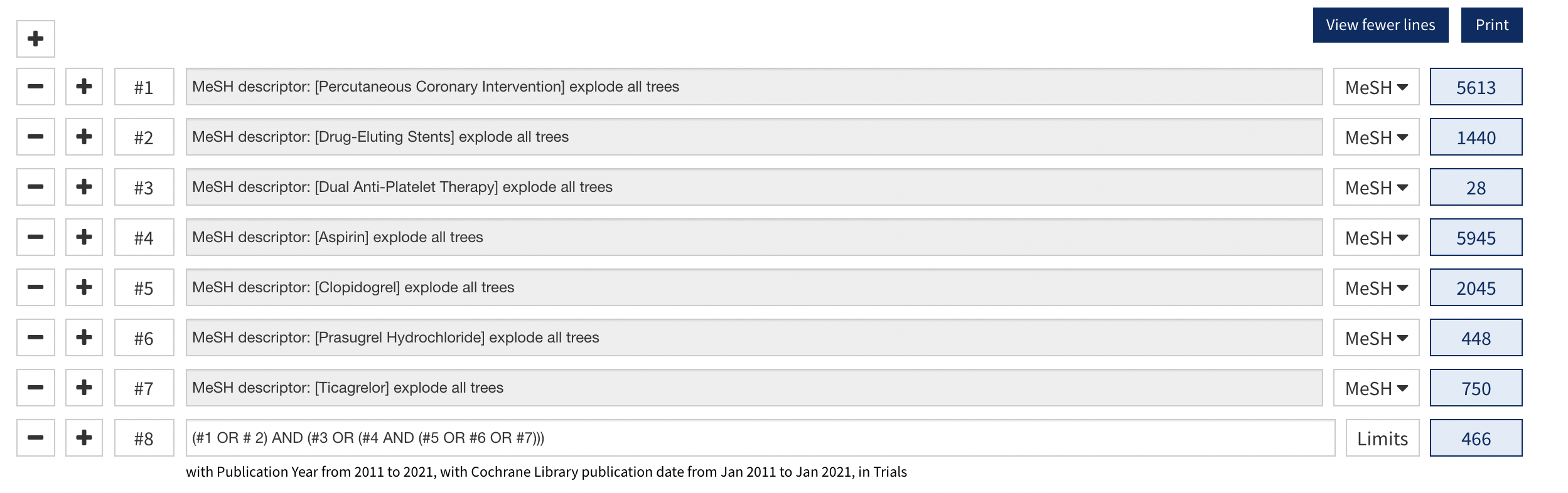


**Embase: (n=1193), we used shortcut keys to select RCTs from the recent 10 years**

('percutaneous coronary intervention'/exp OR 'coronary intervention, percutaneous':ab,ti OR 'coronary interventions, percutaneous':ab,ti OR 'intervention, percutaneous coronary':ab,ti OR 'interventions, percutaneous coronary':ab,ti OR 'percutaneous coronary interventions':ab,ti OR 'percutaneous coronary revascularization':ab,ti OR 'coronary revascularization, percutaneous':ab,ti OR 'coronary revascularizations, percutaneous':ab,ti OR 'percutaneous coronary revascularizations':ab,ti OR 'revascularization, percutaneous coronary':ab,ti OR 'revascularizations, percutaneous coronary':ab,ti OR 'drug eluting stent'/exp OR 'drug-eluting stents':ab,ti OR 'stents, drug-eluting':ab,ti OR 'stents, drug eluting':ab,ti OR 'drug-eluting stent':ab,ti OR 'drug eluting stent':ab,ti OR 'stent, drug-eluting':ab,ti OR 'drug-coated stents':ab,ti OR 'drug coated stents':ab,ti OR 'stents, drug-coated':ab,ti OR 'stents, drug coated':ab,ti OR 'drug-coated stent':ab,ti OR 'drug coated stent':ab,ti OR 'stent, drug-coated':ab,ti

'dual antiplatelet therapy'/exp OR 'anti-platelet therapies, dual':ab,ti OR 'anti-platelet therapy, dual':ab,ti OR 'dual anti platelet therapy':ab,ti OR 'dual anti-platelet therapies':ab,ti) AND ('acetylsalicylic acid'/exp OR 'aspirin':ab,ti OR 'acid, acetylsalicylic':ab,ti OR '2-(acetyloxy)benzoic acid':ab,ti OR 'acylpyrin':ab,ti OR 'aloxiprimum':ab,ti OR 'colfarit':ab,ti OR 'dispril':ab,ti OR 'easprin':ab,ti OR 'ecotrin':ab,ti OR 'endosprin':ab,ti OR 'magnecyl':ab,ti OR 'micristin':ab,ti OR 'polopirin':ab,ti OR 'polopiryna':ab,ti OR 'solprin':ab,ti OR 'solupsan':ab,ti OR 'zorprin':ab,ti OR 'acetysal':ab,ti OR ('clopidogrel'/exp OR 'sc 25989c':ab,ti OR 'sc 25990c':ab,ti OR 'sr 25989':ab,ti OR 'clopidogrel-mepha':ab,ti OR 'clopidogrel mepha':ab,ti OR 'clopidogrel sandoz':ab,ti OR 'iscover':ab,ti OR 'clopidogrel napadisilate':ab,ti OR 'clopidogrel hydrochloride':ab,ti OR 'pcr 4099':ab,ti OR 'pcr-4099':ab,ti OR 'clopidogrel besylate':ab,ti OR 'clopidogrel besilate':ab,ti OR 'clopidogrel, (+)(s)-isomer':ab,ti OR 'plavix':ab,ti OR 'clopidogrel bisulfate':ab,ti AND ('prasugrel'/exp OR 'prasugrel hydrochloride':ab,ti OR 'hydrochloride, prasugrel':ab,ti OR 'prasugrel hcl':ab,ti OR 'hcl, prasugrel':ab,ti OR 'cs 747':ab,ti OR '747, cs':ab,ti OR 'cs-747':ab,ti OR 'cs747':ab,ti OR 'efient':ab,ti OR 'effient':ab,ti OR 'ly 640315':ab,ti OR '640315, ly':ab,ti OR 'ly640315':ab,ti OR 'ly-640315':ab,ti OR 'prasugrel'/exp OR 'prasugrel hydrochloride':ab,ti OR 'hydrochloride, prasugrel':ab,ti OR 'prasugrel hcl':ab,ti OR 'hcl, prasugrel':ab,ti OR 'cs 747':ab,ti OR '747, cs':ab,ti OR 'cs-747':ab,ti OR 'cs747':ab,ti OR 'efient':ab,ti OR 'effient':ab,ti OR 'ly 640315':ab,ti OR '640315, ly':ab,ti OR 'ly640315':ab,ti OR 'ly-640315':ab,ti OR 'ticagrelor'/exp OR 'Brilique':ab,ti OR 'AZD 6140':ab,ti OR 'AZD6140':ab,ti OR 'AZD-6140':ab,ti OR 'Brilinta':ab,ti OR '3-(7-((2-(3,4-Difluorophenyl)cyclopropyl)amino)-5-(propylthio)-3H-(1-3)-triazolo(4,5-d)pyrimidin-3-yl)-5-(2-hydroxyethoxy)cyclopentane-1,2-diol':ab,ti)))

Supplementaryal Table 1.1. Definition of Major Bleeding.

| NO. | Trials | Definition |
| --- | --- | --- |
| 1 | DAPT-STEMI | TIMI Major |
| 2 | EXCELLENT | TIMI Major |
| 3 | ISAR-SAFE | TIMI Major |
| 4 | IVUS-XPL | TIMI Major |
| 5 | OPTIMA-C | TIMI Major |
| 6 | OPTIMIZE | Intracranial, intraocular, or retroperitoneal |
| 7 | SMART-CHOICE | BARC 3-5 |
| 8 | SMART-DATE | BARC 3-5 |
| 9 | TICO | TIMI Major |

BARC, Bleeding Academic Research Consortium; TIMI, Thrombolysis in Myocardial Infarction.

Supplementaryal Table 1.2. Definition of Any Bleeding.

| NO. | Trials | Definition |
| --- | --- | --- |
| 1 | DAPT-STEMI | TIMI |
| 2 | EXCELLENT | TIMI |
| 3 | ISAR-SAFE | TIMI |
| 4 | OPTIMIZE | Modified Major REPLACE 2 & severe or life-threatening GUSTO Criteria |
| 5 | REDUCE | BARC |
| 6 | SMART-CHOICE | BARC |
| 7 | SMART-DATE | BARC |

GUSTO, Global Utilization of Streptokinase and TPA for Occluded arteries.

Supplementary Table 3. Outcomes of subgroup analyses.

| Endpoint | Subgroup | | Trials | HR (95%CI) | P value |
| --- | --- | --- | --- | --- | --- |
| Major bleeding | S-DAPT | 3 months | 3 | 0.65 (0.45, 0.94) | 0.981 |
|  |  | 6 months | 6 | 0.66 (0.37, 1.17) |  |
|  | SAPT | Aspirin | 7 | 0.67 (0.42, 1.08) | 0.855 |
|  |  | P2Y12 RI | 2 | 0.64 (0.42, 0.96) |  |
|  | Patients | ACS | 3 | 0.57 (0.37, 0.87) | 0.356 |
|  |  | CAD | 6 | 0.76 (0.49, 1.18) |  |
| Any bleeding | S-DAPT | 3 months | 3 | 0.71 (0.54, 0.93) | 0.302 |
|  |  | 6 months | 4 | 0.57 (0.43, 0.77) |  |
|  | SAPT | Aspirin | 6 | 0.66 (0.53, 0.82) | 0.634 |
|  |  | P2Y12 RI | 1 | 0.58 (0.36, 0.93) |  |
|  | Patients | ACS | 3 | 0.73 (0.53, 1.01) | 0.33 |
|  |  | CAD | 4 | 0.59 (0.46, 0.77) |  |
| All causes of death | S-DAPT | 3 months | 4 | 1.01 (0.77, 1.33) | 0.229 |
|  |  | 6 months | 7 | 0.78 (0.56, 1.08) |  |
|  | SAPT | Aspirin | 9 | 0.91 (0.71, 1.15) | 0.977 |
|  |  | P2Y12 RI | 2 | 0.91 (0.58, 1.43) |  |
|  | Patients | ACS | 5 | 0.95 (0.70, 1.29) | 0.781 |
|  |  | CAD | 7 | 0.90 (0.67, 1.19) |  |
| Cardiac death | S-DAPT | 3 months | 3 | 0.99 (0.68, 1.46) | 0.379 |
|  |  | 6 months | 6 | 0.76 (0.47, 1.21) |  |
|  | SAPT | Aspirin | 8 | 0.90 (0.65, 1.23) | 0.923 |
|  |  | P2Y12 RI | 1 | 0.86 (0.38, 1.93) |  |
|  | Patients | ACS | 3 | 0.91 (0.57, 1.48) | 0.904 |
|  |  | CAD | 6 | 0.88 (0.60, 1.28) |  |
| Myocardial infarction | S-DAPT | 3 months | 4 | 1.01 (0.75, 1.35) | 0.135 |
|  |  | 6 months | 6 | 1.46 (0.99, 2.15) |  |
|  | SAPT | Aspirin | 8 | 1.29 (1.00, 1.66) | 0.027 |
|  |  | P2Y12 RI | 2 | 0.62 (0.34, 1.13) |  |
|  | Patients | ACS | 5 | 1.24 (0.85, 1.80) | 0.624 |
|  |  | CAD | 6 | 1.10 (0.82, 1.48) |  |
| Definite/Probable stent thrombosis | S-DAPT | 3 months | 4 | 1.41 (0.83, 2.39) | 0.993 |
|  |  | 6 months | 5 | 1.40 (0.79, 2.48) |  |
|  | SAPT | Aspirin | 7 | 1.39 (0.92, 2.11) | 0.884 |
|  |  | P2Y12 RI | 2 | 1.51 (0.54, 4.23) |  |
|  | Patients | ACS | 4 | 1.50 (0.89, 2.52) | 0.717 |
|  |  | CAD | 5 | 1.30 (0.73, 2.32) |  |
| Target-vessel revascularization | S-DAPT | 3 months | 3 | 1.13 (0.86, 1.48) | 0.783 |
|  |  | 6 months | 4 | 1.22 (0.76, 1.94) |  |
|  | SAPT | Aspirin | 6 | 1.18 (0.93, 1.50) | 0.425 |
|  |  | P2Y12 RI | 1 | 0.80 (0.32, 2.01) |  |
|  | Patients | ACS | 3 | 0.95 (0.63, 1.42) | 0.244 |
|  |  | CAD | 4 | 1.27 (0.95, 1.68) |  |
| Stroke | S-DAPT | 3 months | 4 | 1.03 (0.58, 1.82) | 0.927 |
|  |  | 6 months | 5 | 1.07 (0.64, 1.80) |  |
|  | SAPT | Aspirin | 7 | 1.00 (0.63, 1.59) | 0.699 |
|  |  | P2Y12 RI | 2 | 1.18 (0.59, 2.36) |  |
|  | Patients | ACS | 4 | 0.81 (0.47, 1.39) | 0.169 |
|  |  | CAD | 5 | 1.39 (0.80, 2.41) |  |

Supplementary Table 4. Sensitivity analysis.

**Any bleeding**

**Major bleeding**

**All causes of death**

**Cardiac death**

**Myocardial infarction**

**Definite/Probable stent thrombosis**

**Target vessels revascularization**

**Stroke**

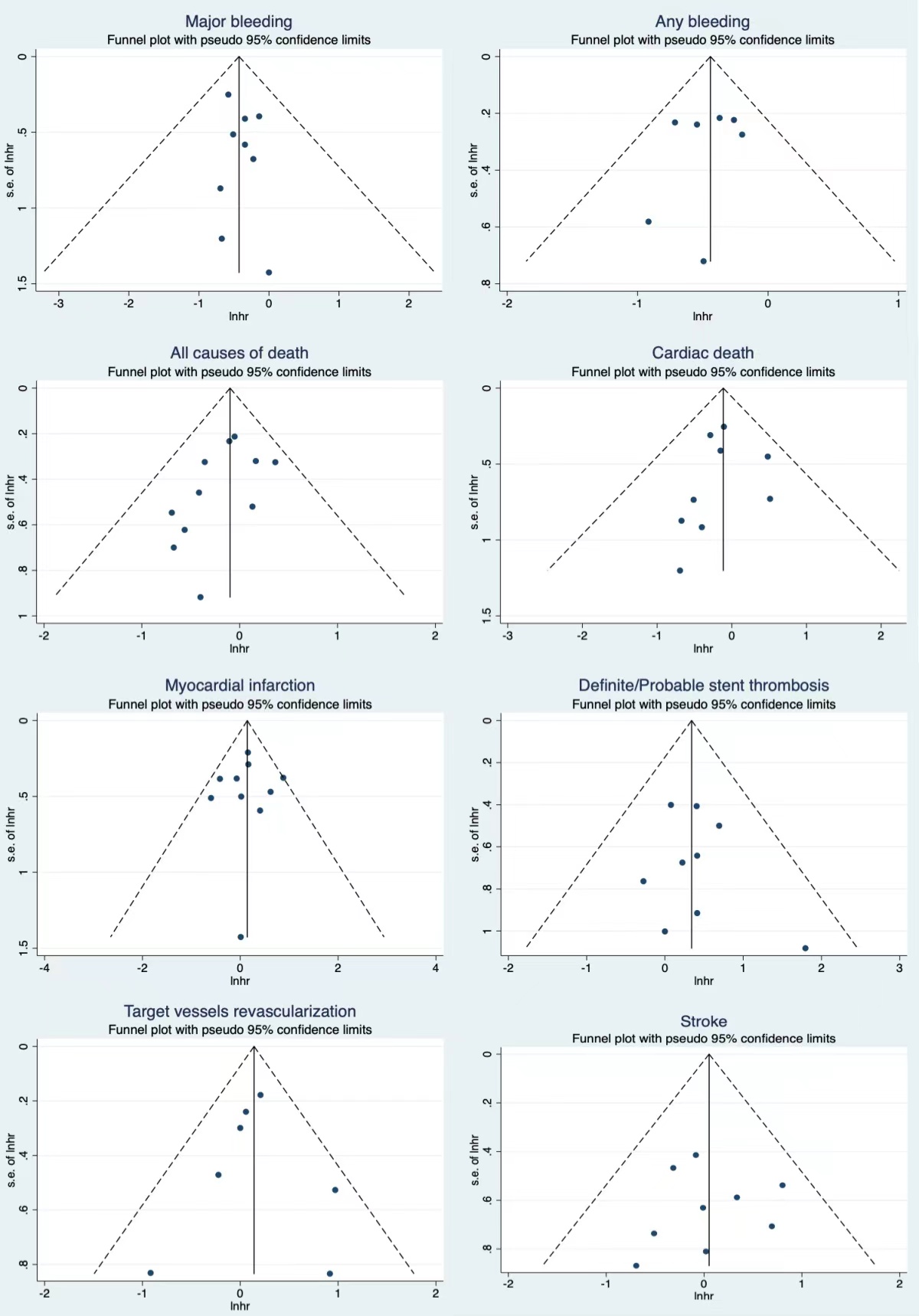


Supplementary Fig. 1. Funnel plot.

Supplementary Table 5. Egger test.

**Any bleeding**

**Major bleeding**

**All causes of death**

**Cardiac death**

**Myocardial infarction**

**Definite/Probable stent thrombosis**

**Target vessels revascularization**

**Stroke**
